# Cognitive assessment: A challenge for occupational therapists in Brazil

**DOI:** 10.1590/1980-57642016dn11-020004

**Published:** 2017

**Authors:** Juliana Conti

**Affiliations:** 1 Occupational Therapy Division of the Hospital das Clínicas/Sao Paulo University, São Paulo, SP, Brazil

**Keywords:** cognitive impairment, occupational therapy, assessment, cognitive assessment, déficit cognitivo, terapia ocupacional, avaliação, avaliação cognitiva

## Abstract

Cognitive impairment is a common dysfunction after neurological injury. Cognitive assessment tools can help the therapist understand how impairments are affecting functional status and quality of life.

**Objective:**

The aim of the study was to identify instruments for cognitive assessment that Occupational Therapists (OT) can use in clinical practice.

**Methods:**

The instruments published in English and Portuguese between 1999 and 2016 were systematically reviewed.

**Results:**

The search identified 17 specific instruments for OT not validated in Brazilian Portuguese, 10 non-specific instruments for OT not validated in Brazilian Portuguese, and 25 instruments validated for Portuguese, only one of which was specific for OT (Lowenstein Occupational Therapy Cognitive Assessment).

**Conclusion:**

There are few assessment cognitive tools validated for use in the Brazilian culture and language. The majority of the instruments appear not to be validated for use by OT in clinical practice.

## Abstract (pt)

Déficits cognitivos são comuns após uma lesão neurológica. Avaliação cognitiva pode auxiliar o terapeuta a compreender melhor as dificuldades do sujeito e como afetam as habilidades funcionais e qualidade de vida.

**Objetivo:**

O objetivo desta pesquisa foi de identificar avaliações cognitivas que o Terapeuta Ocupacional (TO) pode utilizar na prática clínica.

**Métodos:**

Uma revisão sistemática da literatura foi realizada sobre os instrumentos publicados em inglês e português nas bases de dados de 1999 a 2016.

**Resultados:**

Foram identificados 17 instrumentos de avaliação específicos para Terapia Ocupacional, mas que não estão validados em português, 10 instrumentos que não são específicos, mas que podem ser utilizados por TO que também não foram validados para a população brasileira e por fim 24 instrumentos validados, porém apenas um é específico para TO (Lowenstein Occupational Therapy Cognitive Assessment).

**Conclusão:**

Existem poucas avaliações cognitivas validadas para a cultura e língua brasileira. Possivelmente a maioria destes instrumentos não tenha sido validado para a TO utilizar na pratica clínica.

## INTRODUCTION

Cognition is defined as a mental process by which knowledge and understanding is developed in the mind.^1^ In addition, cognition involves the processes of memory, judgment, thinking, reasoning and perception, and has an important role in emotions and behavior.^2^ Cognitive deficits affect activities of daily living (ADL) and instrumental activities of daily living (IADL), leading to disability and loss in quality of life.^2^ Such deficits can also be a barrier to returning to work. Because cognitive impairments are 'invisible', patients have less awareness of them, making it more difficult to recognize the deficits in the workplace and make the necessary adjustments. An integrated approach to patients is the key to identifying compensatory strategies and providing adequate rehabilitation.^2^

The prevalence of cognitive impairment in Brazil was reported in a study conducted in Ribeirão Preto. The study population comprised 1145 adults over 60 years old with heterogeneous conditions, such as stroke, head injury, epilepsy, depression, diabetes, hypertension, cholesterol, arthritis, smoking, alcohol abuse and benzodiazepine use. Out of the 1145 subjects, 217 (18.5%) had cognitive dysfunction.^3^ In another study conducted in the United Kingdom, 15,051 subjects completed the assessment, revealing a prevalence of cognitive impairment of 18.3%.^4^ Moreover, the study showed the influence of cognitive deficits on physical aspects of the patients, who presented the following symptoms: hearing and vision deficits, urinary incontinence and the occurrence of two or three falls in the preceding days.^4^

After brain damage, it is important that the patient begins a rehabilitation process for both physical and cognitive aspects.^5^ There is growing evidence of the benefits of cognitive rehabilitation after brain damage.^5^ Therefore, for effective rehabilitation it is essential to perform an initial assessment to evaluate and understand the cognitive deficits of each patient and inform rehabilitation planning for patients after neurological disease. Numerous cognitive assessment tools are available in the international literature; however, there are few instruments for non-psychologist professionals, such as Occupational Therapists.

The aim of occupational therapy is to help patients develop more independence and autonomy after brain damage. Considering the importance of cognitive aspects during the rehabilitation process, it is essential that the OT be able to evaluate these aspects. Moreover, the OT is thus able to provide rehabilitation for this kind of patient throughout the recovery process.^6^

Before starting rehabilitation, it is essential that the OT carries out an adequate evaluation of the cognitive aspects of the patient. The American Occupational Therapy Association (AOTA) divides instruments into six different types: interview (e.g. Canadian Occupational Performance Measure); cognitive screening tools (e.g. Loewenstein Occupational Therapy Cognitive Assessment); performance-based assessments that may be used to assess cognitive and executive function-based performance deficits once these have been established (e.g. Multiple Errands Test and Árnadóttir OT-ADL Neurobehavioral Evaluation); measures of specific cognitive functions and client factors (e.g. Contextual Memory Test); specific measures of cognitive performance in the context of specific occupations (e.g. Executive Function Performance Test); and environmental assessment (e.g. Home Environmental Assessment Protocol).^7^

The aim of this study was to review the OT literature to find cognitive assessment tools available internationally for individual adults with neurological injury or diseases and compare with instruments available in Brazil.

## METHODS

A sensitive focused literature research strategy was used in this study. Assessments tools were identified by searching the PUBMED, GOOGLE Scholar and GOOGLE books databases for publications between 1989 and December 2016, using the following search terms: occupational therapy, assessment, cognitive assessment and cognitive impairment.

**Inclusion criteria.** 1) Tool with psychometric data; 2) Specific use for OT or non-psychologists; 3) Applicability in individuals with neurologic diseases or brain injury, such as: stroke, traumatic brain injury, brain tumor, multiple sclerosis and dementia; 4) Applicability for age over 18 years; 5) Instruments described in the manuscripts; 6) Instruments in English or translated to Portuguese.

Tools that were not described in detail, and those focused on other diseases, such as mental health, were excluded. Tools cited in original papers, systematic reviews or meta- frequencies of analyses were included. The use of the different evaluation tools across the literature was checked.

Tables 1 to 3 describe the following items: (1) name of the tool; (2) categories: cognitive domains evaluated; and (3) administration time of the tool. The tools are listed in the tables in alphabetical order. Table 1 describes the instruments for occupational therapy practice not validated in Brazil (17 tools); Table 2 shows cognitive assessment tools for use in clinical practice by different health professionals (including occupational therapists) not validated in Brazil (10 tools); and Table 3 shows cognitive assessment tools for use by different health professionals in clinical practice (including occupational therapists) validated in Brazil (25 tools).

**Table 1:** Cognitive assessment tools for OT not validated in Brazil.^22-30^

Tool	Categories	Administration time (approximate)
Arnadottir OT-ADL Neurobehavioural Evaluation (A-ONE).^30^ Divided in two sub-scales	Therapist observes the neurobehavioral of the person performing the Activities of Daily Living	25 minutes
Assessment of Motor and Process Skills^31^	Therapist observes motor and process skills during the performance of ADLExecutive function	Depends on patient's ability and on task chosen.
Activity of Daily Living Profile^32^	Performance on Activities of Daily Living and Instrumental Activities of Daily LivingAssess the executive dysfunctions to perform these tasks	More than one therapy session. Depends on patient's ability.
Chessington OT Neurological Assessment Battery (COTNAB)^33,34^	Visual functions, ability to follow instructions, sensory motor ability, constructional ability	More than 60 minutes.
Cognitive Assessment of Minnesota (CAM)^17^	Memory, attention, orientation, simple math skills, simple money skills, executive function, visuospatial, skills	40 minutes
Cognitive Competency Test (CCT)^35^	Cognitive skills to perform Activities of Daily Living	30-40 minutes
Cognitive Performance Test^36^	Level of cuing and demonstration required	60 minutes
Contextual Memory Test^37^	Memory	10-20 minutes
Executive Function Performance Test^18^	Executive Functions	40 minutes
Execution of a Cooking Task^38^	Prepare two recipes: baking a cake and making an omeletAssess the executive dysfunctions to perform these tasks	60 minutes
Independent Living Scales (ILS)^38^	Memory, orientation, simple money skills, managing home and transportation	45 minutes
Kitchen Task Assessment (part of EFPT)^39^	Executive Functions	20-40 minutes
Ontario Society of Occupational Therapist Perceptual Assessment^40^	Sensation, gnosis, praxis, scanning, body awareness, spatial relation	50-60 minutes
Rivermead Perceptual Assessment Battery (RPAB)^16^	Picture matching, object matching, size recognition, missing article,sequencing-pictures, right/left copying words, color matching, right/left copying shapes, figure-ground, animal halves, body-image	50-60 minutes
The Complex Task Performance Assessment^41^	Executive Functions	30-40 minutes
The Naturalistic Action Test (NAT)^42^	Assess errors during execution of daily routine activitiesExecutive Functions	45-90 minutes
Virtual Action Planning Supermarket (VAP-S)^19^	Simulates real supermarketExecutive Functions	Depends on patient's ability

**Table 2:** Cognitive assessment tools not validated in Brazil that can be used by OT.^22-29^

Tool	Categories	Administration time (approximate)
The Behavioral Inattention Test (BIT)^43^	Unilateral neglect in everyday tasks	40 minutes
Cognitive Abilities Screening Instrument (CASI)^44^	Attention, concentration, memory, language, visual skills, abstraction and judgment	15-20 minutes
Middlesex Elderly Assessment of Mental State (MEAMS)^45^	Orientation, new learning, memory, language, simple math's skills, visuo-spatial skills, perception and motor perseveration	10 minutes
Motor Free Visual Perceptual Test (MVPT) Version 1 or 3^46,47^	Discrimination, figure-ground, visual memory, visuospatial functions, visual closure	20-30 minutes
Severe Impairment Battery (SIB)^49^	Memory, language, orientation, visuospatial, praxis, social interaction	20-30 minutes
Test of Everyday Attention (TEA)^50^	Attention, executive function, visuospatial functions, auditory and visual demands	45-60 minutes
Note: For OT it is necessary to obtain a Thames Valley Test Company endorsed license during one-day course^29^		
The Multiple Errands Test (MET)^50^	Executive Functions	40 minutes
Wessex Head Injury Matrix (WHIM)^51^	Assess and monitor recovery of cognitive function after severe head injury	10-15 minutes
Westmead Post Traumatic Amnesia Scale^27^	Orientation, memory, ability to learn, language, attention	10-15 minutes
Virtual Multiple Errands test^52^	Executive Functions	Depends on patient's ability

**Table 3:** Cognitive assessment tools validated in Brazil that can be used by OT.^24-26,53-55^

Tool	Categories	Administration time (approximate)
Addenbrooke's Cognitive Examination^56^	Memory, orientation, language, praxis, following commands	15-20 minutes
Alzheimer's Disease Assessment Scale^57^	The test is divided into two parts: cognitive assessment (memory, language, praxis and understanding commands) behavior assessment	30-45 minutes
Benton Visual Recognition Test^58^	Evaluates visual memory and visual perception	10-20 minutes
Brief Cognitive Screening Battery^59^	Memory, attention, executive function, visuospatial function, language, simple math ´s skills	30-40 minutes
Cambridge Cognitive Examination-Revised (CAMCOG-R)^60^	Brief cognitive assessment for elderly with cognitive impairment The functions evaluated are memory, language, attention, perception, praxis and thinking	20 minutes
Cancellation task^61,62^	Visuospatial function, sustained and selective attention, psychomotor speed, visual searching and motor coordination	10-15 minutes
Clock Drawing Test^63^	Visuospatial, attention and executive functions	5 minutes
Cognitive Abilities Screening Instrument - Short Form^64^	Evaluates verbal fluency, orientation and recall	30 minutes
Digit Span^65^	This test evaluates working memory	10-15 minutes
Direct Assessment of Functional Status- revised (DAFS-R)^66^	Orientation, Communication, simple money skills, memory and ADL and IADL	30-40 minutes
Executive Interview (EXIT 25)^67^	Executive function and behavior	10 minutes
Frontal Assessment Battery (FAB)^68^	Executive function	30-40 minutes
Fuld Object Memory Evaluation^69^	Evaluates learning and memory in elderly	30 minutes
Functional Assessment Measure (FAM)^11,70^	Must be used in conjunction with the FIMBehavioral, orientation, emotional status, communication, swallowing and community ability	20 minutes
Functional Independence Measure (FIM)^70^	Memory, social interaction, functional status	20-30 minutes
Informant Questionnaire on Cognitive Decline in the Elderly (IQCODE)^71^	This is a short questionnaire to assess cognitive decline in elderly	30 minutes
Loewenstein Occupational Therapy Cognitive Assessment (LOTCA)^13^	Orientation, perception, visuo-motor and thinking operations	30-90 minutes
Mini-Mental State Exam (MMSE)^17^	Memory, orientation, language, praxis, following commands	10-15 minutes
Short Cognitive Performance Test^72^	Cognitive screening to detect memory and attention impairment	30 minutes
SIDAM Portuguese Version^73^	This test is divided into 4 parts: clinical history, cognition, personality and behavior and dementia etiology	30 minutes
Spatial Delayed Recognition Span Task^74^	This is a computerized test that evaluates visuo-spatial working memory	20-30 minutes
The Montreal Cognitive Assessment (MoCA)^12,75^	Language, memory, praxis, attention, orientation, executive function, abstraction and visuospatial	15-20 minutes
Token Test^76^	Language is the main cognitive function evaluated in this test	20-30 minutes
Verbal Fluency^77^	Language and executive functions	5 minutes
Visual Object and Space Perception Battery- VOSP^78^	Visuo-perceptual and visuo-spatial functions	30 minutes

## RESULTS

During the search on PubMed, Google Books and Google Scholar, 12 manuscripts (and instrument sales website to describe these in detail) were selected because they described different types of tools and the application form. From these articles and website, 40 different tools that met the inclusion criteria were included in the review. Table 1 describes cognitive assessments tools (17 instruments) developed by occupational therapist for occupational therapists, not validated in Portuguese. Table 2 shows cognitive instruments developed (10 instruments) for non-psychologists, not validated in Portuguese. Table 3 shows instruments (25 general cognitive assessment tools including 1 specific tool for occupational therapists) validated in Brazil that non-psychologists can use in clinical practice. There was only one cognitive assessment tool specifically developed for occupational therapists and validated for use in the Brazilian population: the Loewenstein Occupational Therapy Cognitive Assessment - LOTCA.

Instruments described in the tables can be specific for one cognitive domain, such as the Executive Dysfunction Performance Test (for executive functions) or for more than one cognitive functions, such as the Cognitive Assessment of Minnesota (memory, attention, orientation, visuospatial, executive functions, reasoning). Each instrument has a different administration time according to the domain and patient difficulties performing the tool task.

Instruments can be divided into: (1) a task to be performed by the patients, where the therapist gives the score according to the tool's rules (e.g. Executive Dysfunction Performance Test, Árnadóttir OT-ADL Neurobehavioral Evaluation, Activity of Daily Living Profile and Execution of a Cooking Task); or (2) a questionnaire/exercise to be completed by patients and scored by the therapist (e.g. Westmead Post Traumatic Amnesia Scale, Addenbrooke's Cognitive Examination and MiniMental State Exam).

When opting to use a specific instrument, it is necessary to learn and practice it before administration to patients. Some of the instruments require a course to start using them, while others can be understood by reading the manual before use (e.g. Executive Dysfunction Performance Test). Moreover, it is essential to determine whether the instrument is appropriate for a specific disease or not, and if it has been validated for the target population.

## DISCUSSION

The Society of Cognitive Rehabilitation reports that in order to provide better rehabilitation for individuals with neurological diseases or injury,^5^ the team of health professionals should comprise doctors, psychologists, occupational therapist, physiotherapists and speech and language therapists. The rehabilitation process is complex and should be performed by the health professional team to achieve patient and family goals.^5^ During rehabilitation planning, aspects of the patient and families must be considered, such as cognitive, emotional, motor aspects of daily routine, social and financial status. Before planning rehabilitation, it is essential to understand the patient's impairments and potential, making it important to carry out an assessment with the appropriate tools.

Different tools for assessing cognitive functions specifically for use in occupational therapy were found by the search. However, only one of these instruments (Loewenstein Occupational Therapy Cognitive Assessment) is validated and adapted for the Brazilian population. Professionals should be able to choose between different types of instrument, according to the patient's needs and clinical practice, because patients are evaluated during the different stages of diseases and injuries and these tools assist during the rehabilitation process. Moreover, different rehabilitation settings (hospital, outpatient clinic, community) require different assessment tools for individuals at different stages of recovery, so different tools are required to provide better understanding and aid planning of rehabilitation. When the health professional decides to use an instrument to evaluate a patient it is essential that the tool is validated for the population target, not only for a given language and culture, but also for the specific disease/injury.^8^

The most commonly reported tools for cognitive assessment are described in Tables 1-3. Most of the instruments are straightforward and can be quickly administered. Although found relatively frequently in the literature evaluating cognitive dysfunction in individuals with neurological diseases, we identified few reports of the validity of these tests for this population in Brazil: Loewenstein Occupational Therapy Cognitive Assessment,^9,10^ Functional Assessment Measure,^11^ The Montreal Cognitive Assessment^12^ and Mini-Mental State Exam.^13,14^

Instruments that require purchase and training for their application, such as the Loewenstein Occupational Therapy Cognitive Assessment,^15^ the Rivermead Perceptual Assessment Battery^16^ and the Cognitive Assessment of Minnesota^17^ were not described in the literature as tools for research, but for use in clinical practice. On the other hand, some instruments are accessible on the internet, such as the Executive Function Performance Test^18^ and Mini-Mental State Exam;^13,14^ however, the manual must be followed during assessment administration and is readily found in the literature and validated in other populations.

Some of the instruments described in the tables are more specific for Dementia (Alzheimer's disease Assessment Scale, Informant Questionnaire on Cognitive Decline in the Elderly, and SIDAM Portuguese Version); however. they can be used for screening cognitive impairment. These types of tools may alert the OT about cognitive impairment and possible need for referral to a specialized professional for assessment and diagnosis. In addition, most of the instruments to assess cognitive decline described in Table 3 are administrated by a neuropsychologist.

Virtual ecological assessment tools , such as the Virtual Action Planning Supermarket - VAP-S^19^ and Virtual Multiple Errands test,^19^ are now more commonly found in the literature because these instruments are suitable for clinical practice and clinical research. They simulate a real environment and demonstrate how the patient should manage in a new situation and in an unfamiliar setting. In the hospital setting or rehabilitation clinics, virtual assessment tools can be very effective because not all patients are allowed to leave their wards for evaluation in a different setting. In addition, these tools may also help to ascertain whether patients with severe impairment will be able to use the computer in their daily routine (communication, cognitive training, groceries shopping, paying bills, leisure, clothes shopping, and even for leisure).

The Cognitive Assessment of Minnesota^17^ is a more complete instrument for Occupational Therapists to evaluate their patients during the initial assessment before planning the rehabilitation process. The Executive Function Performance Test^18^ and Rivermead Perceptual Assessment Battery^16^ are instruments for specific cognitive functions, i.e. these instruments can show the impairments in details.

After an appropriate evaluation, it is time to plan the rehabilitation process for the patient. In case of cognitive rehabilitation after a brain injury or disease, normally we describe patients with brain injury; however, there is a lack of evidence on cognitive rehabilitation and effectiveness.^20^ In another study, the author described evidence for the effectiveness of the treatment of language and perceptions of individuals with traumatic brain injury and stroke.^21^ They also discussed the benefits for treating attention, memory, executive dysfunction, and functional communication in individuals with traumatic brain injury, according to recommendations establishing parameters for effective treatment.^21^

The main limitations of these studies are the low number of studies in this area compared with those on physical dysfunction; a lack of psychometric data for the instruments, especially in Portuguese; and limited evidence to define the best instrument for different diseases or injury and at different stages of recovery.

The limitations of this review were: a lack of instruments validated in Brazil; few studies developed for OT relative to those for other health professionals. As discussed, it is essential to have more than one instrument to choose from when evaluating a patient, because sometimes a specific function is impaired whereas in other cases all cognitive functions need evaluating.

In conclusion, understanding the cognitive impairments begins with a complete evaluation of the patient's deficits. These deficits have an impact on the functional status and quality of life of patients, therefore these impairments should be of concern to all members of the health professional team, including occupational therapists when planning the rehabilitation program. For this reason, is important to define the best instruments for this purpose based on the evidence in the literature.

Despite the lack of instruments specific for OT in Brazil, there are many others tools that can help OT understand the cognitive impairment and how it affects functional status. On the other hand, instruments developed by OT for OT seem to be more effective for clinical practice, due to the intrinsic understanding of how impairments interfere in daily routine activities.

Translation and validation of the instruments for different cultures and languages is essential to help occupational therapists better understand their patients. Further research in this area should be carried out, given the impact of these deficits on the rehabilitation and life of these individuals.
